# Analysis of Variance Combined with Optimized Gradient Boosting Machines for Enhanced Load Recognition in Home Energy Management Systems

**DOI:** 10.3390/s24154965

**Published:** 2024-07-31

**Authors:** Thales W. Cabral, Fernando B. Neto, Eduardo R. de Lima, Gustavo Fraidenraich, Luís G. P. Meloni

**Affiliations:** 1 Department of Communications, School of Electrical and Computer Engineering, University of Campinas, Campinas 13083-852, Brazil; t264377@dac.unicamp.br (T.W.C.); gfraiden@unicamp.br (G.F.); 2 Copel Distribuição S.A., Curitiba 81200240, Brazil; fernando.bauer@copel.com; 3 Department of Hardware Design, Instituto de Pesquisa Eldorado, Campinas 13083-898, Brazil; eduardo.lima@eldorado.org.br

**Keywords:** gradient-boosting machine, LightGBM, HistGBM, XGBoost, machine learning, HEMS, appliance recognition

## Abstract

Load recognition remains not comprehensively explored in Home Energy Management Systems (HEMSs). There are gaps in current approaches to load recognition, such as enhancing appliance identification and increasing the overall performance of the load-recognition system through more robust models. To address this issue, we propose a novel approach based on the Analysis of Variance (ANOVA) *F*-test combined with SelectKBest and gradient-boosting machines (GBMs) for load recognition. The proposed approach improves the feature selection and consequently aids inter-class separability. Further, we optimized GBM models, such as the histogram-based gradient-boosting machine (HistGBM), light gradient-boosting machine (LightGBM), and XGBoost (extreme gradient boosting), to create a more reliable load-recognition system. Our findings reveal that the ANOVA–GBM approach achieves greater efficiency in training time, even when compared to Principal Component Analysis (PCA) and a higher number of features. ANOVA–XGBoost is approximately 4.31 times faster than PCA–XGBoost, ANOVA–LightGBM is about 5.15 times faster than PCA–LightGBM, and ANOVA–HistGBM is 2.27 times faster than PCA–HistGBM. The general performance results expose the impact on the overall performance of the load-recognition system. Some of the key results show that the ANOVA–LightGBM pair reached 96.42% accuracy, 96.27% F_1_, and a Kappa index of 0.9404; the ANOVA–HistGBM combination achieved 96.64% accuracy, 96.48% F_1_, and a Kappa index of 0.9434; and the ANOVA–XGBoost pair attained 96.75% accuracy, 96.64% F_1_, and a Kappa index of 0.9452; such findings overcome rival methods from the literature. In addition, the accuracy gain of the proposed approach is prominent when compared straight to its competitors. The higher accuracy gains were 13.09, 13.31, and 13.42 percentage points (pp) for the pairs ANOVA–LightGBM, ANOVA–HistGBM, and ANOVA–XGBoost, respectively. These significant improvements highlight the effectiveness and refinement of the proposed approach.

## 1. Introduction

Balancing electricity consumption with sustainability is one of the central issues of modern society. Although all participants in the electricity market seek to increase their earnings, as pointed out by Mansouri et al. [1], current solutions in the electricity market have been incorporating more sustainable options such as renewable sources, as discussed in Nie et al. [2] and Mansouri et al. [3]; additionally, the current market is considering the consumer as a prosumer, as indicated by Zhang et al. [4] and Zhou et al. [5], capable of using and supplying energy to the grid, which ensures the prominence of the residential sector. As Cary and Benton [6] pointed out, the residential sector had an energy-saving capacity of more than 66 TWh. In recent reports, as demonstrated by Bang et al. [7] and Rashid et al. [8], this sector accounts for approximately 30% of electricity waste in various manners, such as device inefficiency and unsuitable consumption. One of the promising solutions for efficient consumption comes from Smart Homes (SHs) employing Home Energy Management Systems (HEMSs).

**Figure 1:**
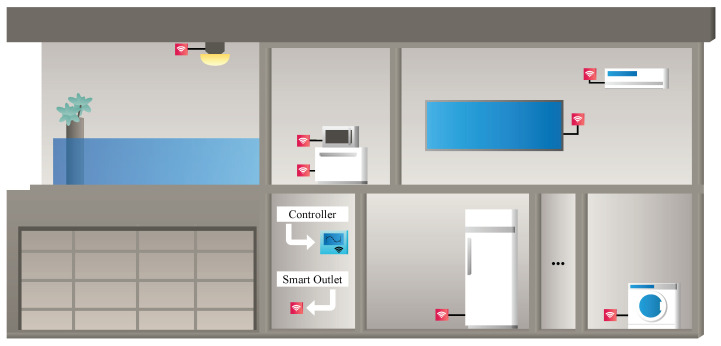
Representation of a typical Home Energy Management System (HEMS).

A HEMS architecture can manage energy usage in residential environments. For this task, the HEMS collects various data with information from the appliances and decides on the management based on this information entanglement. Generally, a HEMS consists of a controller and smart outlets, as illustrated in Figure 1. There is a lot of information that HEMS can monitor about the household appliances’ activity, such as frequency, temperature, active power, and several other types of data. However, in recent discussions, as manifested by Mahapatra and Nayya [9] and Motta et al. [10], a modern architecture ensures added functions for HEMSs, such as load forecasting as in Jo et al. [11], load disaggregation as demonstrated in Lemes et al. [12], appliance anomaly detection as in Tsai et al. [13] and Lee et al. [14], and load recognition as documented in Cabral et al. [15].

The motivation for this study stems from several relevant issues and the broader context of energy management in residential settings. One of the primary challenges faced in the field is the significant amount of electricity waste due to inefficient appliances and unsuitable consumption habits, which accounts for approximately 30% of total residential electricity usage [7,8]. Addressing this waste is critical for achieving sustainability goals and reducing energy consumption. Another challenge is the complexity of accurately identifying the various devices operating simultaneously within a household. Traditional methods often fall short in accuracy and reliability, especially in environments where multiple appliances are active concurrently. The implementation of advanced techniques, as proposed in this study, becomes vital in addressing these challenges. The existing literature explores a variety of solutions for load recognition. Nevertheless, gaps remain. For instance, while techniques like Principal Component Analysis (PCA) can reduce the volume of data for information processing, they may always not preserve the most informative patterns necessary for effective decision-making. The dynamic nature of household energy consumption patterns adds complexity to the load-recognition process, requiring robust and adaptive models, and many current approaches lack the robustness for reliable appliance identification. Addressing these gaps can have significant potential implications, such as efficient working appliance management, reducing energy waste, and improving the overall energy efficiency of residences. The potential benefits extend beyond individual households, contributing to broader societal goals of sustainability and environmental conservation.

However, what is load recognition? As per Faustine and Pereira [16], load recognition is the identifying process of which device is in operation. Why is load recognition relevant? Load recognition plays a vital function in load disaggregation strategies, specifically for appliance identification in the post-disaggregation stage. On the other hand, in the domestic environment, where multiple appliances operate concurrently—such as air conditioners, freezers, heaters, and other devices—it is crucial for HEMSs to discern, in an accurate way, which devices are working, especially when replacing devices connected to smart outlets. Thus, in real-life scenarios, load recognition allows HEMSs to determine automatically the new appliance operating. Likewise, another practical relevance is evident in the automatic building of databases. For dataset creation, through electrical signals analysis, load recognition improves the robustness of database production.

There are several manners to perform load recognition. The most advanced methods use machine learning (ML) techniques. Generally, these approaches incorporate robust strategies for feature handling and employ ML models that demonstrate stability and reliability in decision-making, even under challenging conditions. Currently, works employ diverse approaches to process the features. In Borin et al. [17], the study used the Stockwell transform for feature extraction. Qaisar and Alshari [18] and Soe and Belleudy [19] chose to employ electrical operating patterns from household appliances. In Baets et al. [20], the authors utilized Voltage–Current (VI) trajectories, such as images, to analyze the device patterns. In Zhiren et al. [21] and in Cabral et al. [15], the researchers used the widely known PCA to extract the features. Following this trend at the decision-making stage, more modern studies also explore a variety of techniques. Qaisar and Alshari [18] employ the models Support Vector Machine (SVM) and *k*-Nearest Neighbors (*k*-NN) for appliance identification. On the other hand, besides *k*-NN and SVM, Soe and Belleudy [19] use Classification and Regression Trees (CART), Linear Discriminant Analysis (LDA), Logistic Regression (LR), and Naive Bayes (NB). In Huang et al. [22], the authors apply Long Short-Time Memory Back-Propagation (LSTM-BP) in the classification phase. Furthermore, as presented by Cabral et al. [15], it is possible to employ ensemble methods based on decision trees (DTs), like Random Forest (RF), for load recognition. Although the methods propose diverse ways of processing the features and identifying the appliances, some gaps in the literature remain untapped.

Gaps in approaches to load recognition still exist, such as enhancing appliance identification performance by improving inter-class separability and boosting the overall performance of the load-recognition system through more reliable models. The present work addresses both of the previously mentioned gaps through what we call the ANOVA–GBM approach. Unlike our principal competitor, the work presented by Cabral et al. [15], which uses PCA to process the features aiming to improve inter-class separability, we used the Analysis of Variance (ANOVA) *F*-test with SelectKBest to enhance the inter-class separability. PCA is a feature extraction technique that projects the characteristics of the data into another feature space that may have reduced dimensionality. However, this projection is not always enough to preserve the most informative patterns that feed the decision-making model. To address this issue, we propose a feature selection technique in which the most informative patterns are chosen by applying the ANOVA *F*-test with SelectKBest, thus avoiding the necessity of a forced projection of the data. We also introduce gradient-boosting machine (GBM) architectures in load recognition to ensure higher reliability for appliance identification at the decision-making stage. GBM approaches are ensemble architectures that combine multiple models to produce a more robust final model, where the intention is to correct the errors made by the prior model or set of previous models. In addition to our propositions to deal with the gaps, the proposed approach includes other strategies that make it a robust system for load recognition, such as data preprocessing to determine the ON/OFF appliance state, a procedure to determine the optimal number of features via Cumulative Explained Variance (CEV), and grid search (GS) with K-fold cross-validation (K-CV) to optimize the chosen GBM, contributing to the model generalization capacity. The results of our approach, based on ANOVA–GBM, show the highest accuracy values, weighted average F_1_-Score, and Kappa index in comparison with the competitors’ strategies from the literature. It is relevant to mention that our solution is part of an ongoing research project called Open Middleware and Energy Management System for the Home of the Future. This initiative is a collaboration between the University of Campinas, the Brazilian energy company Copel Distribuição S.A. (Curitiba, Brazil), and the Eldorado Research Institute.

### Principal Contributions

The principal contributions of our work consist of the following:

1. **Novel approach to load recognition:** our study proposes a pioneering approach to load-recognition systems based on the ANOVA *F*-test with SelectKBest and GBMs. This research is the first to use the ANOVA *F*-test with SelectKBest to recognize loads in HEMSs, which improves the feature selection and, consequently, aids inter-class separability. This characteristic improves system performance, as enhanced separability ensures that GBMs can more efficiently differentiate the classes. Furthermore, this work is the first to apply GBMs such as the histogram-based gradient-boosting machine (HistGBM), light gradient-boosting machine (LightGBM), and XGBoost (extreme gradient boosting) for load recognition in HEMS applications. Employing robust models like GBMs results in higher reliability for the load-recognition system. Due to this original proposal, this paper also presents a pioneering analysis of the ANOVA–HistGBM, ANOVA–LightGBM, and ANOVA–XGBoost combinations for the task of load recognition;
2. **Practical implications:** the ANOVA–GBM approach achieves greater efficiency in training time, even when compared to PCA for a higher number of features. It should be noted that ANOVA–XGBoost is approximately 4.31 times faster than PCA–XGBoost, ANOVA–LightGBM is about 5.15 times faster than PCA–LightGBM, and ANOVA–HistGBM is 2.27 times faster than PCA–HistGBM. In addition, the results show that the ANOVA–GBM approach achieves the highest values for accuracy, weighted average F_1_-Score, and Kappa index—96.75%, 96.64%, and 0.9452, respectively—compared to competing strategies in the literature. These practical implications are driven by the enhanced feature selection capability and the use of more robust and reliable models, leading to significant improvements in the performance of the load-recognition system and demonstrating the effectiveness and refinement of the proposed approach;
3. **Advances in the load-recognition field:** in addition to significantly enhancing the performance and efficiency of load-recognition systems, our study contributes to fundamental elements present in load-recognition systems, such as data preprocessing, feature handling, machine learning architectures, optimization methodologies, level of intrusibility, and reliability. Additionally, this study exploits remaining gaps in load-recognition approaches, such as improving appliance identification performance by enhancing feature selection and boosting the overall performance of the load-recognition system through more reliable models. Notably, the ANOVA *F*-test with SelectKBest and GBM models establishes a new standard for feature selection and ML architectures in load-recognition systems. This advancement fosters the development of more robust, accurate, and reliable systems, positively impacting academic research and practical applications in the home energy management sector;
4. **Bibliographic survey of contemporary load-recognition systems:** we offer a bibliographic survey of contemporary load-recognition systems, concentrating on key aspects such as data preprocessing, feature processing, machine learning architectures, optimization techniques, degree of intrusiveness, and reliability. This review provides insights into the latest advancements in load-recognition technology, addressing crucial components that determine system performance and usability.

The structure of the remaining sections is outlined as follows: Section 2 presents detailed background to contextualize this study. Section 3 offers a meticulous description of the proposed system, detailing its processing flow. This section displays the approach to feature selection, criteria for feature relevance, and the optimization of machine learning models. Section 4 introduces the metrics employed in this study, alongside their rationale, and examines the results obtained with the proposed approach. Additionally, this section analyzes the findings and offers new insights. Section 5 presents the manuscript conclusion, evaluating the implications of the proposed strategy, highlighting key findings, and identifying promising aspects of the proposed system.

## 2. Theoretical Background

This section provides a state-of-art review of the load-recognition approaches and presents the foundational concepts, encompassing feature selection via the ANOVA *F*-test with SelectKBest and the architecture of the GBMs.

### 2.1. Related Works

In this section, we provide an bibliographic review of modern load-recognition systems, with a focus on fundamental elements including data preprocessing, feature extraction, machine learning architectures, optimization methodologies, level of intrusiveness, and reliability. Considering related works, this review provides insights into the latest advancements in load-recognition technology.

- **Data preprocessing:** preprocessing, including state detection (ON-OFF), is not mandatory for load-recognition strategies. However, this procedure enables the detection of appliance activities. Consequently, it is possible to determine if a device is functioning or turned off. Only the most recent techniques incorporate a preprocessing strategy capable of discerning whether an appliance is in operation. As presented in Table 1, in Matindife et al. [23], the authors employed an RMS threshold for this task. Meanwhile, in Cabral et al. [15] and our proposed system, the Discrete Wavelet Transform (DWT) was utilized to detect the operational status of appliances.
- **Feature processing:** undoubtedly, feature processing is one of the most relevant stages in load recognition. As listed in Table 1, current approaches employ numerous feature-processing techniques. In Matindife et al. [23], researchers applied the Gramian Angular Difference Field (GADF) for feature extraction. In Borin et al. [17], the authors utilized the Stockwell transform for the same task. Meanwhile, the study by Heo et al. [24] incorporates information from Amplitude–Phase–Frequency (APF). Approaches by Qaisar and Alsharif [18] and Soe and Belleudy [19] are grounded in the extraction of operating patterns from the active power of appliances. Consistent with Soe and Belleudy [19], the work presented by Mian Qaisar and Alsharif [25] also relies on operating patterns, albeit considering both the active and reactive power of appliances. Zhiren et al. [21] additionally include harmonics generated by appliances in their analysis, combining them with active and reactive power to produce 2D clusters. Following a similar path, De Baets et al. [20] demonstrate the feasibility of developing a feature processing stage utilizing the VI trajectories, composed of 2D frames as images, capable of characterizing appliance behavior. However, Faustine and Pereira [16] advocate for decomposing signal characteristics into components using the Fryze power theory. The works of Huang et al. [22] and Cabral et al. [15] recommend the application of PCA, which decomposes relevant data information into components. Alternatively, we propose pioneeringly employing the ANOVA *F*-test with SelecKBest to select the most relevant features.
- **Machine learning architecture:** in the literature, several ML architectures can be suitable for the decision-making stage, where the architecture can distinguish the type of equipment in operation. Architectures based on Artificial Neural Networks (ANNs) are highly adaptable to data, meaning that they can easily adjust to the data. The studies by Zhiren et al. [21] explored the use of the Extreme Learning Machine (ELM) and AdaBoost-ELM, while Mian Qaisar and Alsharif [25] investigated the application of ANNs. Leveraging the ability to extract relevant features from structured and unstructured data, De Baets et al. [20], Faustine and Pereira [16], and Matindife et al. [23] proposed the use of CNNs. Huang et al. [22] introduced the Long Short-Time Memory Back-Propagation (LSTM-BP) model, which combines the properties of long-term memory with the back-propagation algorithm. Heo et al. [24] employed the Hilbert Transform Long Short-Term Memory (HT-LSTM), whose hybrid architecture combines the Hilbert Transform and the Long Short-Time Memory module. However, because ANN-based models can excessively learn the data, they are more susceptible to overfitting, which may reduce their generalization capacity. On the other hand, traditional models also have their place. The studies by Mian Qaisar and Alsharif [25], Soe and Belleudy [19], Qaisar and Alsharif [18], and Cabral et al. [15] explored the application of *k*-NN for this task. Meanwhile, the studies by Zhiren et al. [21], Soe and Belleudy [19], Qaisar and Alsharif [18], and Cabral et al. [15] highlighted the application of SVMs. Additionally, Soe and Belleudy [19] employed tree-based models such as CART for the first time, while Cabral et al. [15] investigated the application of the widely known decision tree (DT). However, as highlighted in Table 1, only in Cabral et al. [15] was a tree ensemble model, the RF, considered in load recognition. RF is an ensemble, but it is not a GBM. At this stage, the present work proposes pioneering alternatives for GBMs: HistGBM and LightGBM.
- **Optimization procedure:** beyond ML architecture, it is necessary to ensure more stable and reliable model estimates. High performance and robustness in decision-making stage are requirements present in the most modern load-recognition strategies. To ensure this, Table 1 highlights that only Cabral et al. [15] and our proposed system employ grid search with K-fold cross-validation.
- **Level of intrusibility:** most HEMSs can collect a wide variety of data, including active power, reactive power, frequency, voltage, current, and many other parameters. However, leveraging such a comprehensive dataset is not always advantageous. With each additional parameter explored, the load-recognition strategy becomes more complex, heightening its level of intrusibility. This manuscript introduces a nuanced categorization of intrusiveness, delineated into three tiers to address this issue: low, intermediate, and high, as outlined in Table 1. The high level employs an excessive variety of parameters, as exemplified by the paper by Huang et al. [22], which utilizes more than three parameters, i.e., steady-state power, the amplitude of the fundamental wave of the transient current, transient voltage, and the harmonics of the transient current. An intermediate level incorporates more than one piece of information up to three, such as in the study by De Baets et al. [20], which utilizes voltage and current, and the work of Zhiren et al. [21], which employs active power, reactive power, and harmonics. A low level signifies that the approach utilizes only one piece of information to feed the system; for example, in Cabral et al. [15] and in our system, we employ only active power.
- **Reliability:** most investigations do not assess the reliability of their strategies. In some cases, authors only employ a single performance metric, such as the most common one, accuracy. To honestly evaluate the system’s reliability, it is necessary to apply metrics that consider the performance of the models holistically. Some works, such as Cabral et al. [15] and Matindife et al. [23], listed in Table 1, utilize the Kappa index as a metric dedicated to the agreement between the predicted and expected outcomes reached by the system. However, this is underexplored by Matindife et al. [23]. Meanwhile, the work of Cabral et al. [15] demonstrates a high level of reliability, surpassed only by our proposed system.

**Table 1:** List of state-of-the-art approaches and their specificities.

Load-Recognition Systems	Data Preprocessing	Feature Processing	Machine Learning Architecture	Optimization Procedure	Level of Intrusibility	Reliability
Huang et al. [22]	None	PCA	LSTM-BP	None	High	Not reported
De Baets et al. [20]	None	VI trajectories	CNN	None	Intermediate	Not reported
Faustine and Pereira [16]	None	Analysis of high-frequency properties	CNN	None	Intermediate	Not reported
Mian Qaisar and Alsharif [25]	None	Analysis of consumption patterns	ANN and *k*-NN	None	Intermediate	Not reported
Zhiren et al. [21]	None	Analysis of electrical quantity	ELM, AdaBoost-ELM, and SVM	None	Intermediate	Not reported
Matindife et al. [23]	None	GADF	CNN	None	Intermediate	Barely explored
Heo et al. [24]	Yes/RMS Threshold	APF	HT-LSTM	None	Intermediate	Not reported
Borin et al. [17]	None	Stockwell transform	VPC	None	Low	Not reported
Soe and Belleudy [19]	None	Analysis of operating patterns	CART, *k*-NN, LDA, LR, NB, and SVM	None	Low	Not reported
Qaisar and Alsharif [18]	None	Analysis of operating patterns	*k*-NN and SVM	None	Low	Not reported
Cabral et al. [15]	Yes/DWT	PCA	DT, *k*-NN, RF, and SVM	Grid search with K-fold cross-validation	Low	High reliability
Our System	Yes/DWT	ANOVA *F*-test with SelectKBest	GBM: HistGBM, LightGBM, and XGBoost	Grid search with K-fold cross-validation	Low	Superior reliability

### 2.2. Feature Selection Using ANOVA *F*-Test with SelectKBest

As presented by Sthle and Wold [26], the ANOVA *F*-test is a powerful statistical technique for comparing means across multiple groups and identifying significant differences. In the context of feature selection, the ANOVA *F*-test procedure assesses the significance of features inherent to the data. However, to effectively reduce data dimensionality, de facto, a method is necessary to evaluate this significance. Regarding this, the ANOVA *F*-test can determine the statistical significance of each feature. The ANOVA *F*-test is based on *F* scoring. *F* scoring is a statistical test to compare the variances of two populations using the ratio between the variances. As per Prasad et al. [27], a high scoring value indicates that the means of the features are significantly different, which suggests that these features can better separate the categories. Following the formal exposition by Prasad et al. [27], we can apply *F* with the aid of two variables: the between-group variance ($\sigma_{\beta }^{2}$) and within-group variance ($\sigma_{w}^{2}$). Therefore, we can calculate $F=\sigma_{\beta }^{2}/\sigma_{w}^{2}$, where $\sigma_{\beta }^{2}=\sum_{i=1}^{G}n_{i}{\left({\bar{S}}_{i}-\bar{S}\right)}^{2}/\left(G-1\right)$ and $\sigma_{w}^{2}=\sum_{i=1}^{G}\sum_{j=1}^{n_{i}}{\left(S_{ij}-{\bar{S}}_{i}\right)}^{2}/\left(L-G\right)$. As per Prasad et al. [27], *L* epresents the total sample size, *G* denotes the number of groups, $n_{i}$ expresses the number of observations in the *i*th group, $S_{ij}$ represents the *j*th observation in the *i*th out of *G* groups, $\bar{S}$ denotes the overall mean of the variable set and ${\bar{S}}_{i}$ represents the sample mean of the *i*th group. In the sequel of the ANOVA *F*-test, it is necessary to select the most relevant characteristics. As per Raufi and Longo [28], the SelectKBest method allows the selection of the *k* best features based on a ranking. When combined with the ANOVA *F*-test, SelectKBest evaluates all features and selects the features with the highest scores.

### 2.3. Gradient Boosting Machines (GBMs)

**Figure 2:**
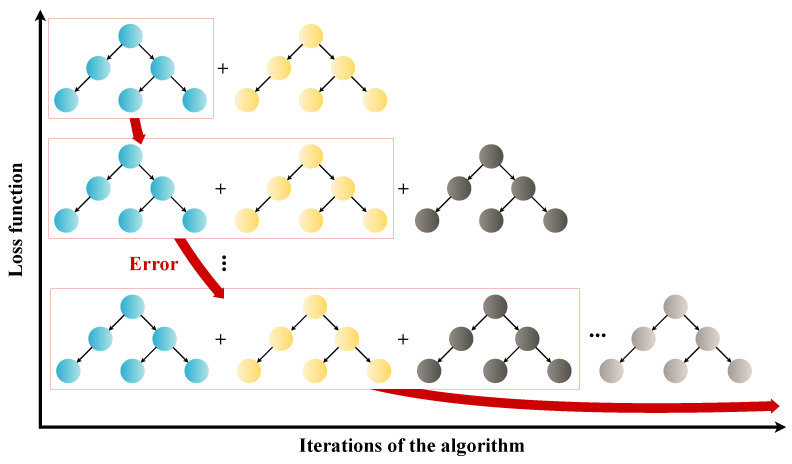
Representation of the tree-building algorithm (adapted from Nhat-Duc and Van-Duc [30]).

The term boosting refers to a class of algorithms based on ensemble learning, in which the algorithms sequentially add trees to the collection. According to Rufaida et al. [29], tree construction runs as the ensemble error decreases, as illustrated in Figure 2. This class of algorithms allows training to be made faster depending on the construction of each tree, although the algorithms can be slow for large datasets. For this reason, an elegant solution is to propose treating the data beforehand through a dimensionality reduction technique, which can guarantee excellent results such as, in our case, the proposed use of the ANOVA *F*-test with SelectKBest.

As per the formal description of GBMs, in accordance with Louk and Tama [31], considering a dataset D with x inputs, containing *k* features with *m* instances and *y* labels, we can define D = $\left\{\left(x_{i},y_{i}\right)\;|\;i\in \left\{1,\ldots ,m\right\},\;x_{i}\in R^{k},\;y_{i}\in R\right\}$. Next, we can consider the prediction output as $\sum_{i=1}^{\tau }g_{i}\left(x\right)$, where $g_{i}$ is the output of *i*-th tree in the τ-tree collection. In the sequence, the algorithm can build the (τ+1)-th tree by minimizing the objective function $\min\left\{O{\left(g\right)}^{t}\right\}=\min\left\{L{\left(g\right)}^{t}+R{\left(g\right)}^{t}\right\},$ where *t* represents the index of the set of trees in an ensemble of trees, and $L{\left(g\right)}^{t}$ and $R{\left(g\right)}^{t}$ are the loss and regularization functions, respectively. The first function, $L{\left(g\right)}^{t}$, calculates the difference between the predicted ${\hat{y}}_{i}$ and the real-value $y_{i}$, i.e., the error. The second function, $R{\left(f\right)}^{t}$, is the regularization function to avoid overfitting and can manage some hyperparameters, for example, number of leaves or additional leaf growth. More details about GBMs can be found in Louk and Tama [31] and Ong et al. [32]. In our study, we consider the general hyperparameter set of any GBM as $\left\{h_{1}^{\left(\mathrm{GBM},k\right)},h_{2}^{\left(\mathrm{GBM},k\right)},\ldots ,h_{J}^{\left(\mathrm{GBM},k\right)}\right\}$ = ${\left\{h_{i}^{\left(\mathrm{GBM},k\right)}\right\}}_{i=1}^{J}$, where *i* is the index of the possible candidate $h_{i}^{\left(\mathrm{GBM},k\right)}$ and $h_{J}^{\left(\mathrm{GBM},k\right)}$ is the last candidate. However, each GBM can contain its specific set of adjustable hyperparameters, depending on the algorithm implementation. This work applies three GBM architectures: HistGBM, LightGBM, and XGBoost. XGBoost and LightGBM, for example, have adjustable hyperparameters such as max depth and the number of estimators. In contrast, the HistGBM algorithm implementation allows for adjusting hyperparameters like max depth and max leaf nodes. Specific details regarding the design of the algorithms LightGBM, XGBoost, and HistGBM can be found in the works by Ke et al. [33], Chen and Guestrin [34], and Nhat-Duc and Van-Duc [30], respectively.

## 3. Proposed System: ANOVA–GBM Approach for Load Recognition

**Figure 3:**
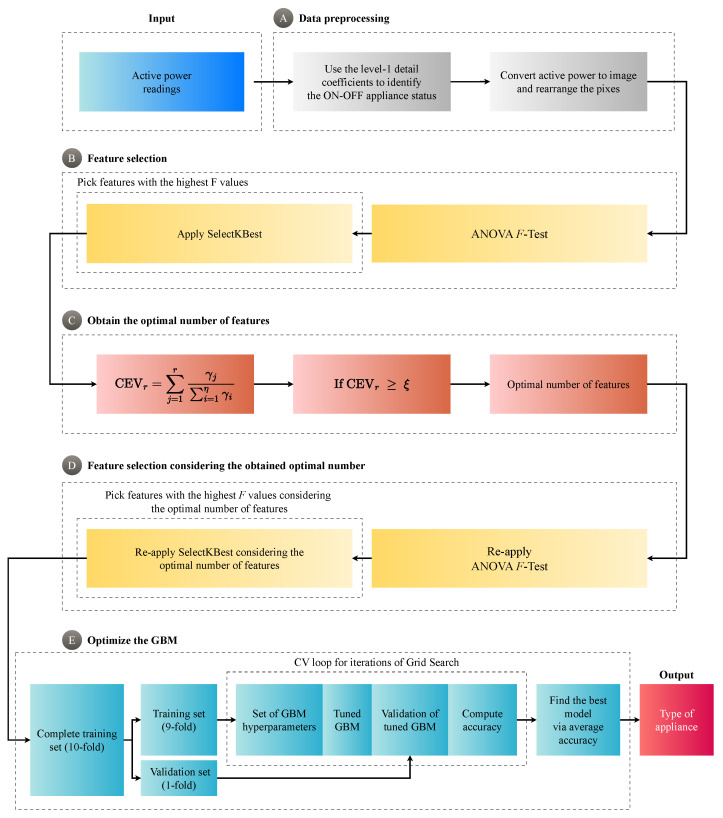
Approach for load recognition focus in Home Energy Management Systems. This diagram outlines the stages of the proposed methodology, beginning with (**A**) the initial preprocessing phase involving the detection of appliances’ ON-OFF status and data processing. Following this, (**B**) the system utilizes feature selection through the ANOVA *F*-test with SelectKBest. Henceforward, (**C**) the CEV is applied to determine the most suitable number of features. In the next stage, (**D**) the system employs feature selection utilizing ANOVA *F*-test with SelectKBest based on the optimal number of features. In the final stage, (**E**) the strategy optimizes the GBM models to enhance classifier performance and ultimately identify the type of operational appliance.

Figure 3 illustrates all the processing chains comprising the designed system. Several processing chains comprise it, beginning with the collected active power from appliances. For data collection, the system uses the Reference Energy Disaggregation Dataset (REDD) from Kolter and Johnson [35], which provides comprehensive power usage data from various household appliances over eight days, registered at a frequency of 1/3 Hz. The REDD dataset provides data collection from Household 1 with a wide variety of appliances, which includes the following devices: oven, refrigerator, dishwasher, kitchen oven, lighting, washer, dryer, microwave, bathroom Ground Fault Interrupters (GFIs) outlet, heat pump, stove, and unknown devices. From the appliances in Household 1, our system generated 4609 images with a resolution of 32×32 pixels (1024 features in total), a sufficient quantity to assess the robustness of the proposed approach. This was followed by feature selection, determination of the optimal number of features, and selection based on that number. Subsequently, the system performed an optimization of the GBM models, culminating in the final output, which identifies the type of appliance.

According to Figure 3A, the system incorporates a preprocessing stage responsible for detecting the ON/OFF states and generating images from active power. For this task, our system employs the Discrete Wavelet Transform (DWT) in the same manner as Lemes et al. [36]. This preprocessing involves the application of the DWT to the active power data to identify the operational states of the appliances. Here, the system uses the level-1 detail coefficients obtained with the Daubechies 4 mother wavelet applied to the active power of the household appliances in the HEMS. The coefficients extracted by the DWT allow for the observation of transition instants between OFF–ON and ON–OFF states through the higher magnitude peaks, where these peaks indicate the beginning and end of the appliance cycles. Afterward, the system converts the identified activity segments into images, following the method outlined by Cabral et al. [15]. Each resulting image captures a cycle of the appliance activity. The system translates the electrical activity curve of the appliance into black pixels on a white background, i.e., the electrical activity curve is converted into black pixels on a white background, creating a visual representation of the appliance operating. The resolution of these images is adjustable, and, for our experiments, the system used a resolution of 32×32 pixels, resulting in 1024 features per image. Next, the method produces a set of *m* images with *k* pixels, arranging this set into a matrix D with dimensions k×m. According to the proposed Algorithm 1, these data, D, are then divided into training and testing sets with a ratio of 80% for training and 20% for testing, following the partition suggested by Géron [37]. The training set is used for hyperparameter tuning and model training, while the testing set is reserved for evaluating the final model performance. Figure 3 summarizes this processing flow, where the load-recognition system solely utilizes the active power gathered from appliances, depicted by the light blue color (Input) in Figure 3A. Subsequently, the data preprocessing is depicted in light gray in Figure 3A.

Figure 3B shows the initial feature selection stage in yellow, where we apply the ANOVA *F*-test with SelectKBest. Algorithm 1 begins by dividing the generated data D into training $D_{\left(p.\mathrm{train}\right)}$ and testing $D_{\left(p.\mathrm{test}\right)}$ sets, adhering to a specified proportion. This step is vital in many machine learning processes to ensure the testing of the trained model and accurate validation of its predictions on previously unseen data. After dividing the dataset, the algorithm applies the ANOVA *F*-test with SelectKBest on the training set $D_{\left(p.\mathrm{train}\right)}$ using an initial number of components (η). As there are no restrictions for the η value because η acts as an initial assumption in Algorithm 1, the initial number of features η = 1024, i.e., the maximum number of features. The ANOVA *F*-test with SelectKBest helps in selecting more informative features. In this step, the selected data then serve as the foundation for determining the optimal number of features in the next stage.
**Algorithm 1** Approach for load recognition based on the Analysis of Variance *F*-test with SelectKBest and the model optimization of the gradient-boosting machines**Input:** Generated dataset (D), proportion of training data (p.train), proportion of test data (p.test), initial number of components (η), threshold (ξ), number of folds (K), set of *J* candidates for the values of the maximum depth hyperparameter of the chosen model/GBM: $\left\{h_{1}^{\left(\mathrm{GBM},k\right)},h_{2}^{\left(\mathrm{GBM},k\right)},\ldots ,h_{J}^{\left(\mathrm{GBM},k\right)}\right\}$ = ${\left\{h_{i}^{\left(\mathrm{GBM},k\right)}\right\}}_{i=1}^{J}$.**Output:** Type of load in operation  1:First step:Divide the D database between $D_{\left(p.\mathrm{train}\right)}$ training set and the $D_{\left(p.\mathrm{test}\right)}$ test set.  2:Second step:Employ the ANOVA with SelectKBest using $D_{\left(p.\mathrm{train}\right)}$ and η initial number of features. In the sequel, obtain the selected data $D_{\left(p.\mathrm{train},\mathrm{ANOVA}\right)}^{\left(\eta \right)}$.  3:Third step:Compute the covariance matrix $C_{\left(\mathrm{ANOVA}\right)}$ from $D_{\left(p.\mathrm{train},\mathrm{ANOVA}\right)}^{\left(\eta \right)}$. We calculate the covariance matrix based on Lemes et al. [12].  4:Fourth step:Obtain the eigenvalues $\gamma_{i}$ via $C_{\left(\mathrm{ANOVA}\right)}=\Lambda \cdot \Gamma \cdot \Lambda^{-1}$, in wich Λ is the eigenvector matrix and Γ is the diagonal matrix, i.e., $diag(\gamma_{1},\gamma_{2},\ldots ,\gamma_{\eta })$.  5:Fifth step:Sort the eigenvalues in descending order: $\gamma_{1}\geq \gamma_{2}\geq \gamma_{3}\geq \ldots \geq \gamma_{\eta }$  6:Sixth step:Discover the optimal number of features (*k*) through CEV:    Generate the variable *k* and set its value to zero    Compute CEV_*r*_=$\sum_{j=1}^{r}\frac{\gamma_{j}}{\sum_{i=1}^{\eta }\gamma_{i}}$        **if** CEV_*r*_≥ξ            *k* ← number of *r*-th feature        **end if**  7:Seventh step:Employ the ANOVA with SelectKBest, according to *k* features, to obtain the new selected data for the training set, i.e., the $D_{\left(p.\mathrm{train},\mathrm{ANOVA}\right)}^{\left(k\right)}$.  8:Eighth step:Employ the possible values for the hyperparameters for each *k*, i.e., ${\left\{h_{i}^{\left(\mathrm{GBM},k\right)}\right\}}_{i=1}^{J}$  9:Ninth step:Apply GS with K-CV    Divide $D_{\left(p.\mathrm{train},\mathrm{ANOVA}\right)}^{\left(k\right)}$ in K folds    Train the model on each K fold    Calculate accuracy    Measure average accuracy    Assign the average accuracy to the current possible values for the hyperparameters    Adopt the hyperparameter with the highest average accuracy achieved: $h_{\mathrm{optimal}}^{\left(\mathrm{GBM},k\right)}$10:Tenth step:Train the chosen GBM with $h_{\mathrm{optimal}}^{\left(\mathrm{GBM},k\right)}$11:Eleventh step:Testing the optimized model: GBM using $D_{\left(p.\mathrm{test},\mathrm{ANOVA}\right)}^{\left(k\right)}$**return** Type of load in operation

Subsequently, as highlighted in orange in Figure 3C, according to Algorithm 1, the system computes the covariance matrix, the $C_{\left(\mathrm{ANOVA}\right)}$, from the data selected by the ANOVA *F*-test with SelectKBest, i.e., from $D_{\left(p.\mathrm{train},\mathrm{ANOVA}\right)}^{\left(\eta \right)}$. After obtaining the eigenvalues from the covariance matrix, the algorithm sorts them in descending order to determine the optimal number of features *k* using Cumulative Explained Variance (CEV). This procedure identifies the minimum number of features that retain most of the original data variability, ensuring a balance between dimensionality reduction and information preservation.

Upon determining the optimal number of features, the algorithm re-applies the ANOVA *F*-test with SelectKBest using only this number of features, with reduced dimensionality, both for the training and testing sets. This ensures that the dataset is now reduced to the most informative features, simplifying the model without significant loss of information. This stage is depicted in Figure 3D, represented in yellow, and Algorithm 1 presents this procedure through the seventh step/method.

In the final stage, Algorithm 1 applies cross-validation using grid search with K-fold to optimize the GBM’s hyperparameters, ensuring that the model achieves maximum robustness. In line with Kuhn et al. [38], we used a 10-fold cross-validation, which means splitting the dataset into ten parts, and the model is trained and validated ten times, each time using a different part as the validation set and the remaining parts as the training set. This processing chain is depicted in Figure 3E, highlighted in light green. The proposed approach employs the grid search to exhaustively search for the best hyperparameters by analyzing different hyperparameter combinations, ensuring optimal performance. The GBM model is then trained with the optimized hyperparameters and tested using the selected test dataset, i.e., the test set. It is worth noting that, in this manuscript, we evaluated three GBM architectures: the XGBoost, the LightGBM, and the HistGBM. Finally, the algorithm outputs the operational load type, depicted in Figure 3 in red (Output), which is the primary objective of the modeling. This meticulous sequential procedure ensures that the final model is well tuned and achieves high levels of robustness and reliability.

## 4. Results and Discussions

Our work does not merely propose an innovative approach but also commits to evaluating its robustness and reliability. Consequently, it is vital to employ multiple metrics in performance evaluation. This manuscript uses three distinct metrics: accuracy, weighted average F_1_-Score (F_1_), and the Kappa index. All these metrics are widely known in the literature. Here, it is pertinent to highlight that each metric offers a unique perspective on the performance of ML models, contributing to a comprehensive inspection. As per Laburú et al. [39], accuracy is essential for overall performance analysis. In our manuscript, accuracy evaluates the overall success rate of the model. We applied accuracy as per Sellami and Rhinane [40] and Lemes et al. [12]. On the other hand, according to Guo et al. [41], F_1_ can provide a subtle analysis of the model performance, especially in situations where class imbalance can exist. Because F_1_ incorporates this effect, we employed F_1_ as one of the evaluation metrics. We applied such a metric following Alswaidan and Menai [42]. In addition, it is necessary to analyze the reliability of the system. As outlined by Matindife et al. [23], Kappa can infer the agreement of the system. In this manner, we can verify the reliability of the proposed approach. The Kappa statistic operates from −1 up to 1. A value of −1 indicates no agreement, 0 signifies agreement by chance, and 1 denotes total agreement. We employed Kappa according to Sellami and Rhinane [40] and Cabral et al. [15].

For the results analysis, this study employed one of the most relevant and widely utilized datasets in the load recognition literature, from Kolter and Johnson [35], the REDD. As highlighted in the table of comparison to other approaches, the REDD is commonly used in the performance evaluation of state-of-the-art approaches. The REDD dataset provides data from eight days of collection from Household 1. According to Kolter and Johnson [35] and Cabral et al. [15], the active power of appliances is registered at a frequency of 1/3 Hz. Additionally, this dataset features a wide variety of appliances, particularly in Household 1, which includes the following devices: oven, refrigerator, dishwasher, kitchen oven, lighting, washer dryer, microwave, bathroom GFIs, outlet, heat pump, stove, and unknown devices. From the appliances in Household 1, our system generated 4609 images with a resolution of 32 × 32 pixels (1024 features in total), a sufficient quantity to assess the robustness of the proposed approach. It should be noted that the system used 4609 images with a resolution of 32×32 pixels, which resulted in 32×32=1024 features. It is pertinent to mention that we did not reduce the number of samples, meaning that the number of images (4609) remained unchanged. Additionally, the images consisted of the electrical activity of the appliances, which are the active power curves that characterize the ON state of each appliance. However, our approach selects the most relevant features, reducing the number of features. Thus, the system reduced the 1024 features to a smaller number, considering the most relevant ones through the proposed approach. It is worth saying that Section 3 and Algorithm 1 detail the procedure for obtaining these relevant features. In line with Géron [37], 80% of the total images ($D_{80\%}$) were allocated for training and 20% ($D_{20\%}$) for testing, with only the training data used for hyperparameter search. For all ML architectures in Algorithm 1, we used K = 10 in the hyperparameter search. As discussed in Kuhn et al. [38], this value provides test error rate estimates without being affected by improper bias or high variance. In addition, we employed the initial number of features η = 1024, i.e., the maximum number of features. There are no restrictions for the η value because η acts as an initial assumption. This choice was not critical because Algorithm 1 determines the suitable number of features *k*. As per Algorithm 1, the system uses CEV and ξ to impose a feasible value of *k*. In simulations, we analyzed different values for *k*, such as 32, 64, 128, 256, 512, and 1024 (by adjusting the values of ξ). However, to surpass the competitor, Cabral et al. [15], we needed ξ = 0.999999 and our system found CEV_*r*_ = 0.99999985, where *k* = 512. It is worth pointing out that the feature selection required more components and, consequently, a higher threshold. On the other hand, to maintain computational efficiency and performance reliability, while the system ran the hyperparameter search, Algorithm 1 applied ${\left\{h_{i}^{\left(\mathrm{GBM},512\right)}\right\}}_{i=1}^{10}$ = $\left\{h_{1}^{\left(\mathrm{GBM},512\right)}=10,h_{2}^{\left(\mathrm{GBM},512\right)}=20,\ldots ,h_{10}^{\left(\mathrm{GBM},512\right)}=100\right\}$ in each GBM hyperparameter search, i.e., ${\left\{h_{i}^{\left(\mathrm{GBM},512\right)}\right\}}_{i=1}^{10}$ = ${\left\{h_{i}^{\left(\mathrm{XGBoost},512\right)}\right\}}_{i=1}^{10}$ = ${\left\{h_{i}^{\left(\mathrm{HistGBM},512\right)}\right\}}_{i=1}^{10}$ = ${\left\{h_{i}^{\left(\mathrm{LightGBM},512\right)}\right\}}_{i=1}^{10}$.

As depicted in Figure 3E, the proposed system applies grid search with K-fold cross-validation to determine the optimal XGBoost hyperparameters. To perform this procedure, Algorithm 1 uses the values of ${\left\{h_{i}^{\left(\mathrm{XGBoost},512\right)}\right\}}_{i=1}^{10}$ for the max depth search and the same values to define the number of estimators. At the end of this procedure, Algorithm 1 finds the optimal max depth and the optimal number of estimators. At this stage, Algorithm 1 found the optimal max depth of 29 and the optimal number of estimators corresponding to 30. In other words, Algorithm 1 determined that the optimal hyperparameter pair $h_{\mathrm{optimal}}^{\left(\mathrm{XGBoost},512\right)}$ = {29,30}. In this scenario, Table 2 lists the average results using optimized XGBoost in 50 runs. When comparing the performance gain between techniques—the Δ—the accuracy gain between ANOVA and PCA achieves an advantage of 0.87 percentage points for ANOVA, which means $\Delta_{\mathrm{acc}}$ = 0.87 pp. For the F_1_, this difference increases, with a gain of 1.03 percentage points, i.e., $\Delta_{F_{1}}$ = 1.03 pp. Upon examining the agreement index, the Kappa, we observe 1.42 percentage points, symbolizing $\Delta_{\mathrm{Kappa}}$ = 1.42 pp. By checking Table 2, ANOVA reaches the highest accuracy, F_1_, and Kappa values—96.75%, 96.64%, and 0.9452, respectively.

Employing LightGBM, Algorithm 1 uses the values of ${\left\{h_{i}^{\left(\mathrm{LightGBM},512\right)}\right\}}_{i=1}^{10}$ for the search of hyperparameters. During this phase, the system found the pair $h_{\mathrm{optimal}}^{\left(\mathrm{LightGBM},512\right)}$ = {20,100}. Table 3 shows the average results from 50 iterations with the optimized LightGBM. By measuring performance gains, the proposed approach achieves $\Delta_{\mathrm{acc}}$ = 1.07 pp. for the accuracy gain, $\Delta_{F_{1}}$ = 1.17 pp. for the F_1_ gain, and $\Delta_{\mathrm{Kappa}}$ = 1.87 pp. for the Kappa gain. In Table 3, ANOVA achieves the highest accuracy, F_1_, and Kappa values—96.42%, 96.27%, and 0.9404, respectively—once again.

In this latter scenario, Algorithm 1 employed HistGBM with the values ${\left\{h_{i}^{\left(\mathrm{HistGBM},512\right)}\right\}}_{i=1}^{10}$ for hyperparameter tuning and, consequently, found the optimal value for the max depth and optimal value for the max number of leaf nodes. In this process, the system identified the optimal parameters as $h_{\mathrm{optimal}}^{\left(\mathrm{HistGBM},512\right)}$ = {20,20}. Evaluating performance enhancements reported in Table 4, the proposed approach results in an accuracy improvement of $\Delta_{\mathrm{acc}}$ = 0.96 pp., an F_1_ increase of $\Delta_{F_{1}}$ = 1.00 pp., and a Kappa enhancement of $\Delta_{\mathrm{Kappa}}$ = 1.61 pp. As shown in Table 4, the ANOVA method consistently achieves the highest values in accuracy, F_1_, and Kappa—96.64%, 96.48%, and 0.9434, respectively.

Another interesting aspect is the training time, for which Table 5 lists the average training for the approaches. Comparing the training times of the strategies, ANOVA–XGBoost presents a training time of 3.67 s, saving approximately 76.77% of the time compared to PCA–XGBoost, which requires 15.80 s. This means that ANOVA–XGBoost is approximately 4.31 times faster than PCA–XGBoost. Similarly, ANOVA–LightGBM, with a time of 10.22 s, saves about 80.57% of the time compared to the PCA–LightGBM technique, which takes 52.61 s, making ANOVA–LightGBM approximately 5.15 times faster than PCA–LightGBM. Finally, the ANOVA–HistGBM technique, with a time of 29.79 s, reduces the training time by about 55.97% compared to the PCA–HistGBM, which requires 67.65 s, making ANOVA–HistGBM approximately 2.27 times faster than PCA–HistGBM. Thus, the ANOVA–XGBoost, ANOVA–LightGBM, and ANOVA–HistGBM approaches are more efficient in terms of training time compared to their respective counterparts.

The load-recognition methods presented in Table 6 vary in their technical approaches, each combining different feature processing strategies and machine learning models to achieve their objectives. For instance, PCA is employed in various methods, such as those by Huang et al. [23] and Cabral et al. [15], due to its ability to reduce data dimensionality without losing crucial information. However, as depicted in Table 6, there is no definitive approach to feature processing. The authors also employ VI trajectories, GADF, Stockwell transform, APF, and consumption pattern analysis. In this context, the proposed approach innovatively employs the ANOVA *F*-test with SelectKBest for feature processing, effectively selecting features that enhance classification performance.

On the other hand, researchers employ a wide diversity of machine learning models in load recognition. Table 6 shows various architectures, such as LSTM-BP and HT-LSTM, which handle sequential data as variations of recurrent neural networks for device identification. Additionally, many methods, including those by De Baets et al. [20] and Matindife et al. [23], frequently use CNNs for automatic feature extraction from complex data. The Artificial Intelligence (AI) models encompass a comprehensive range, including *k*-NN, DT, RF, AdaBoost-ELM, and SVM. In this context, our proposed system leads the way in utilizing GBMs, thereby ensuring both robust performance and high reliability.

As highlighted by Table 6, evaluation metrics vary from F_1_-Score, precision, and accuracy to the Kappa index, providing a comprehensive view of model performance across different contexts. However, only the works of Matindife et al. [23], Cabral et al. [15], and ours employ a dedicated metric for system agreement evaluation, the Kappa index. In addition, there is no consensus regarding the employed dataset. On the other hand, REDD is the most commonly used dataset to evaluate approaches developed by researchers, particularly in more contemporary studies. This dataset offers a rich diversity of appliances and a substantial dataset size, facilitating thorough analysis.

Comparing the performance of the methods, it is evident that each approach has limitations, reaching different values. Huang et al. [22] utilize PCA with LSTM-BP, achieving an F_1_-Score of 45.49% on the REDD dataset, whereas De Baets et al. [20] employ VI trajectories with a CNN, yielding an F_1_-macro of 77.60% on the Plug Load Appliance Identification Dataset (PLAID). Conversely, Borin et al. [17] utilize the Stockwell transform with VPC, reaching 90.00% accuracy on a private dataset. More recent methods, such as those by Cabral et al. [15], employ PCA with different models (*k*-NN, DT, RF, and SVM) and, as listed in Table 6, achieve accuracies starting from 93.49% on the REDD dataset. Our pioneering method employs GBMs reaching the highest accuracies, 96.42% with LightGBM, 96.64% with HistGBM, and 96.75% with XGBoost.

Based on the data presented in Table 6, our proposed method demonstrates noteworthy improvements in accuracy over other approaches. The highest accuracy previously reported is 96.31% by Cabral et al. [15] using PCA and SVM on the REDD dataset. Our method, which utilizes the ANOVA *F*-test with SelectKBest for feature processing and XGBoost for classification, achieves an accuracy of 96.75%. This gain represents an improvement of 0.44 percentage points. Compared to the next highest accuracies, such as 95.40% by Qaisar and Alsharif [18] with SVM and 94.80% by Zhiren et al. [21] with AdaBoost-ELM, our method shows enhancements of 1.35 and 1.95 percentage points, respectively. Overall, our approach results in a performance increase, especially when compared to strategies that use a CNN by Faustine and Pereira [16], achieving 94.00%, *k*-NN by Soe and Belleudy [19], achieving 94.05%, and PCA with DT by Cabral et al. [15], achieving 94.14%. The improvements in these cases are 2.75, 2.70, and 2.61 percentage points, respectively. Although employing different databases, when compared to methods using a CNN by Matindife et al. [23], achieving 83.33%, VPC by Borin et al. [17], achieving 90.00%, and HT-LSTM by Heo et al. [24], achieving 90.04%, the improvements are 13.42, 6.75, and 6.71 percentage points, respectively.

On the other hand, our proposed ANOVA–HistGBM method achieves an impressive accuracy of 96.64%. This result represents a gain of 0.93 percentage points compared to Cabral et al. [15], which achieves 95.71%. Compared with Qaisar and Alsharif [18] who reach 95.40% and Zhiren et al. [21] at 94.80%, our ANOVA–HistGBM method shows improvements of 1.24 and 1.84 percentage points, respectively. Furthermore, our method reveals progress compared to the CNN by Faustine and Pereira [16] with 94.00%, *k*-NN by Soe and Belleudy [19] with 94.05%, and PCA with DT by Cabral et al. [15] with 94.14%, showcasing enhancements of 2.64, 2.59, and 2.50 percentage points, respectively. When compared to methods using a CNN by Matindife et al. [23] at 83.33%, VPC by Borin et al. [17] at 90.00%, and HT-LSTM by Heo et al. [24] at 90.04%, our ANOVA–HistGBM method exhibits gains of 13.31, 6.64, and 6.60 percentage points, respectively.

When applying the ANOVA *F*-test with SelectKBest and LightGBM, our method achieves an accuracy of 96.42%. This performance marks an increase of 0.11 percentage points over the highest accuracy reported by Cabral et al. [15]. When compared to Qaisar and Alsharif [18], which achieves 95.40%, and Zhiren et al. [21], which reaches 94.80%, our method exhibits improvements of 1.02 and 1.62 percentage points, respectively. Furthermore, our approach also demonstrates gains when compared to the CNN by Faustine and Pereira [16], which achieves 94.00%, *k*-NN by Soe and Belleudy [19], which achieves 94.05%, and PCA with DT by Cabral et al. [15], which achieves 94.14%. These comparisons show improvements of 2.42, 2.37, and 2.28 percentage points, respectively. The method also outperforms the CNN by Matindife et al. [23], achieving 83.33%, VPC by Borin et al. [17], achieving 90.00%, and HT-LSTM by Heo et al. [24], achieving 90.04%, with improvements of 13.09, 6.42, and 6.38 percentage points, respectively. Finally, these results underscore the efficacy of our approach in achieving superior accuracy across a diverse set of benchmark comparisons. We believe that performance increases of the proposed method, compared to the direct rival approach, are because of the feature selection technique, which naturally chooses the most significant features.

## 5. Conclusions

In this work, we proposed ANOVA–GBM, which utilizes the ANOVA *F*-test with SelectKBest to enhance feature selection, thereby improving pattern identification between classes, and employs GBM models to achieve a more reliable load-recognition system. Numerical results indicate that the ANOVA–GBM approach achieved the highest values of accuracy, F_1_, and Kappa index compared to other strategies in the literature. The ANOVA–LightGBM combination reached 96.42% accuracy, 96.27% F_1_, and a Kappa index of 0.9404; the ANOVA–HistGBM combination achieved 96.64% accuracy, 96.48% F_1_, and a Kappa index of 0.9434; and the ANOVA–XGBoost combination attained 96.75% accuracy, 96.64% F_1_, and a Kappa index of 0.9452. These prominent results become even more evident when comparing the proposed approach with previous works. For each mentioned pair, our method achieved significant improvements. For the ANOVA–LightGBM pair, the method outperformed HT-LSTM by Heo et al. [24], VPC by Borin et al. [17], and the CNN by Matindife et al. [23], with improvements of 6.38, 6.42, and 13.09 pp in accuracy, respectively. When compared to methods using HT-LSTM by Heo et al. [24], VPC by Borin et al. [17], and a CNN by Matindife et al. [23], our ANOVA–HistGBM approach showed gains of 6.60, 6.64, and 13.31 pp in accuracy, respectively. The improvements for the ANOVA–XGBoost pair compared to HT-LSTM by Heo et al. [24], VPC by Borin et al. [17], and the CNN by Matindife et al. [23] were 6.71, 6.75, and 13.42 pp, respectively. Furthermore, the XGBoost, LightGBM, and HistGBM models with ANOVA exhibited significantly shorter training times when compared to using PCA, even with a higher number of features. The ANOVA–HistGBM pair was 2.27 times faster than PCA–HistGBM, ANOVA-XGBoost was approximately 4.31 times faster than PCA–XGBoost, and ANOVA–LightGBM was about 5.15 times faster than PCA–LightGBM. These results underscore the improvement in the load-recognition system compared to previous works, highlighting the effectiveness and refinement of the proposed approach.

## Figures and Tables

**Table 2:** Employing optimized XGBoost as the GBM in decision-making.

Approaches	Average Accuracy	Average F_1_	Average Kappa
ANOVA–XGBoost	96.75%	96.64%	0.9452
PCA–XGBoost	95.88%	95.61%	0.9310

**Table 3:** Employing optimized LightGBM as the GBM in decision-making.

Approaches	Average Accuracy	Average F_1_	Average Kappa
ANOVA–LightGBM	96.42%	96.27%	0.9404
PCA–LightGBM	95.35%	95.10%	0.9217

**Table 4:** Employing optimized HistGBM as the GBM in decision-making.

Approaches	Average Accuracy	Average F_1_	Average Kappa
ANOVA–HistGBM	96.64%	96.48%	0.9434
PCA–HistGBM	95.68%	95.48%	0.9273

**Table 5:** Average training time in seconds.

**ANOVA–XGBoost**	**ANOVA–LightGBM**	**ANOVA–HistGBM**
3.67 s	10.22 s	29.79 s
**PCA–XGBoost**	**PCA–LightGBM**	**PCA–HistGBM**
15.80 s	52.61 s	67.65 s

**Table 6:** Comparison to other approaches.

Approaches	Feature Processing	Machine Learning Model for the Result	All Evaluation Metrics	Dataset	Best Result for the Dataset
Huang et al. [22]	PCA	LSTM-BP	F_1_-Score	REDD	45.49% of F_1_-Score
De Baets et al. [20]	VI trajectories	CNN	F_1_-macro, precision, and recall	PLAID	77.60% of F_1_-macro
Matindife et al. [23]	GADF	CNN	Accuracy, precision, recall, F_1_-Score, and Kappa	Private	83.33% of accuracy
Borin et al. [17]	Stockwell transform	VPC	Identification percentage	Private	90.00% of accuracy
Heo et al. [24]	APF	HT-LSTM	Accuracy and F_1_-Score	PLAID	90.04% of accuracy
Cabral et al. [15]	PCA	*k*-NN	Accuracy, F_1_, and Kappa	REDD	93.49% of accuracy
Faustine and Pereira [16]	Analysis of high-frequency properties	CNN	F_1_-eb and F_1_-macro	PLAID	94.00% of F_1_-macro
Soe and Belleudy [19]	Analysis of operating patterns	*k*-NN	Accuracy	ACS-F1	94.05% of accuracy
Cabral et al. [15]	PCA	DT	Accuracy, F_1_, and Kappa	REDD	94.14% of accuracy
Cabral et al. [15]	PCA	RF	Accuracy, F_1_, and Kappa	REDD	94.36% of accuracy
Mian Qaisar and Alsharif [25]	Analysis of consumption patterns	ANN	Accuracy	ACS-F2	94.40% of accuracy
Zhiren et al. [21]	Analysis of electrical quantity	AdaBoost-ELM	Accuracy	Private	94.80% of accuracy
Qaisar and Alsharif [18]	Analysis of operating patterns	SVM	Accuracy	ACS-F2	95.40% of accuracy
Cabral et al. [15]	PCA	SVM	Accuracy, F_1_, and Kappa	REDD	96.31% of accuracy
Our System	ANOVA	LightGBM	Accuracy, F_1_, and Kappa	REDD	96.42% of accuracy
Our System	ANOVA	HistGBM	Accuracy, F_1_, and Kappa	REDD	96.64% of accuracy
Our System	ANOVA	XGBoost	Accuracy, F_1_, and Kappa	REDD	96.75% of accuracy

## Data Availability

The database used is openly available at [35].

## Abbreviations

The following abbreviations are used in this manuscript:

ACS-F1	Appliance Consumption Signature-Fribourg 1
ACS-F2	Appliance Consumption Signature-Fribourg 2
AI	Artificial Intelligence
APF	Amplitude–Phase–Frequency
ANN	Artificial Neural Network
ANOVA	Analysis of Variance
CART	Classification and Regression Tree
CEV	Cumulative Explained Variance
CNN	Convolutional Neural Network
DWT	Discrete Wavelet Transform
DT	Decision tree
ELM	Extreme Learning Machine
GADF	Gramian Angular Difference Field
GFI	Ground Fault Interrupter
GS	Grid search
HEMS	Home Energy Management System
HT-LSTM	Hilbert Transform Long Short-Term Memory
K-CV	K-fold cross-validation
*k*-NN	*k*-Nearest Neighbors
LDA	Linear Discriminant Analysis
LR	Logistic Regression
LSTM	Long Short-Time Memory
LSTM-BP	Long Short-Time Memory Back-Propagation
ML	Machine learning
NB	Naive Bayes
PCA	Principal Component Analysis
PLAID	Plug Load Appliance Identification Dataset
ELM	Extreme Learning Machine
RF	Random Forest
REDD	Reference Energy Disaggregation Dataset
RMS	Root Mean Square
SVM	Support Vector Machine
VPC	Vector Projection Classification
